# Room-Temperature Sputtered SnO_2_ as Robust Electron Transport Layer for Air-Stable and Efficient Perovskite Solar Cells on Rigid and Flexible Substrates

**DOI:** 10.1038/s41598-019-42962-9

**Published:** 2019-05-06

**Authors:** Matthew Kam, Qianpeng Zhang, Daquan Zhang, Zhiyong Fan

**Affiliations:** 1 HKUST-Shenzhen Research Institute, No. 9 Yuexing first RD, South Area, Hi-tech Park, Nanshan, Shenzhen 518057 China; 2 Department of Electronic and Computer Engineering, Hong Kong University of Science and Technology (HKUST), Clear Water Bay, Kowloon, Hong Kong SAR China

**Keywords:** Solar cells, Electronic devices

## Abstract

Extraordinary photovoltaic performance and intriguing optoelectronic properties of perovskite solar cells (PSCs) have aroused enormous interest from both academic research and photovoltaic (PV) industry. In order to bring PSC technology from laboratory to market, material stability, device flexibility, and scalability are important issues to address for vast production. Nevertheless, PSCs are still primarily prepared by solution methods which limit film scalability, while high-temperature processing of metal oxide electron transport layer (ETL) makes PSCs costly and incompatible with flexible substrates. Here, we demonstrate rarely-reported room-temperature radio frequency (RF) sputtered SnO_2_ as a promising ETL with suitable band structure, high transmittance, and excellent stability to replace its solution-processed counterpart. Power conversion efficiencies (PCEs) of 12.82% and 5.88% have been achieved on rigid glass substrate and flexible PEN substrate respectively. The former device retained 93% of its initial PCE after 192-hour exposure in dry air while the latter device maintained over 90% of its initial PCE after 100 consecutive bending cycles. The result is a solid stepping stone toward future PSC all-vapor-deposition fabrication which is being widely used in the PV industry now.

## Introduction

Perovskite solar cells (PSCs) as a promising and emerging photovoltaic (PV) technology have quickly caught enormous attention in both academic research and PV industry over the past few years. As one of the most intensively researched types of solar cells, PSCs have been rapidly developed with unprecedented success, leading to a significant improvement on power conversion efficiency (PCE) from 3.8% to certified 22.7% within a decade^1,2^. Besides high device performance, low fabrication cost as well as tunable composition and bandgap give this group of semiconductors gigantic potential and intriguing optoelectronic properties for next-generation solar cell^3^. Crystal growth optimization, interfacial engineering, compositional optimization, and device architecture design have been further explored to enhance PCE and stability of devices and eventually make them competitive with conventional silicon and other leading thin film solar cell technologies^4–8^.

Typically, a PSC consists of an electron transport layer (ETL), a perovskite film and a hole transport layer (HTL), in addition to top and bottom electrical contacts. Particularly, inorganic metal-oxide films, such as TiO_2_ and ZnO, have been widely reported as effective ETLs for high-performance PSCs^9–14^. Nevertheless, they both have major drawbacks. TiO_2_ as the most commonly used ETL has low electron mobility and requires sintering at high temperature up to 500 °C^15–19^. High temperature processing not only increases the cost of device fabrication, but also restricts the compatibility with flexible substrates. In addition, TiO_2_ provokes perovskite degradation under exposure of UV illumination which is problematic during the prolonged device operation^20^. On the other hand, the hygroscopic nature of ZnO easily causes perovskite decomposition in moisture environment. Moreover, ZnO has poor thermal stability so it can easily react with perovskite during annealing and thermal treatment at elevated temperature, which eventually stimulates undesired perovskite degradation or even decomposition. Like perovskite, TiO_2_ and ZnO are mostly prepared by solution methods, which sacrifice film uniformity and limit large-scale device production. On the other hand, SnO_2_ is regarded as an alternative to replace TiO_2_ and ZnO as an effective ETL to achieve low-cost PSCs with improved stability^21,22^. SnO_2_ has a wider bandgap and its electron mobility is two orders of magnitude higher than that of TiO_2_, making it a more suitable candidate for use in high performance devices^23^. Compare to TiO_2_ and ZnO, SnO_2_ is less hygroscopic in nature, has better thermal and UV stability, and possesses lower photocatalytic activity^24^. These properties prevent perovskite degradation and benefit PSC long-term stability. Despite that high-performance PSCs based on SnO_2_ have been achieved, almost all reported SnO_2_ films were prepared by spin-coating^25–30^, atomic layer deposition (ALD)^31,32^, plasma-enhanced ALD^33^, sol-gel process^34^, chemical bath deposition^35^, hydrothermal process^36^, and electrodeposition^37^. In fact, many of these methods involve high-temperature processing and annealing ranging from 100 °C up to 550 °C, which again increases fabrication complexity and cost and makes it incompatible with flexible substrates. In order to make PSC technology cost-effective in the future, a fabrication technique allowing vast production is absolutely necessary.

Considering the high reliability, maturity, and capability for large-scale production of sputtering technique in both industries and laboratories, SnO_2_ prepared by magnetron sputtering for PSC application is however rarely reported^38^. And previously studied sputtered SnO_2_ film was calibrated based on deposition time instead of thickness, which is a more accurate and reliable approach in principle since deposition time depends on a number of deposition parameters and equipment infrastructure. Moreover, previous study lacked investigation on device stability as well as thorough thin film characterization on their SnO_2_ and the corresponding glovebox-processed spin-coated perovskite absorber, particularly morphology and crystallinity analysis by scanning electron microscopy (SEM), X-ray diffraction (XRD), and photoluminescence (PL). Therefore, it was hard to convince the effectiveness and compatibility of sputtered SnO_2_ with perovskite solar cells. On the other hand, perovskite films are typically prepared by solution methods inside a glovebox filled with inert gas. Similarly, those solution methods limit film scalability while the use of glovebox increases production cost and complexity. Researchers have therefore put a great amount of effort to fabricate perovskite films and devices in ambient condition without sacrificing film quality, device performance, as well as stability^19,39^.

Here, we demonstrate room-temperature RF sputtered SnO_2_ film as an effective and robust ETL and meanwhile take one step forward to implement it together with vapor-deposited perovskite absorber for air-stable and efficient PSCs on both rigid and flexible substrates. By this application, we are now only one step away from sputtered SnO_2_ based all-vacuum-deposited perovskite solar cells which will eventually enable industrialization. To the best of our knowledge, there is no report of using sputtered SnO_2_ for flexible PSCs application. Both SnO_2_ thickness and deposition conditions including working pressure and gas environment were systematically investigated. Device stability and hysteresis were also carefully studied.

## Results and Discussion

**Figure 1:**
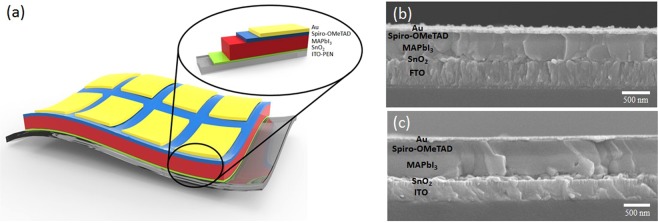
Device architecture and thin film characterization. (**a**) Device architecture of the ITO-PEN/SnO_2_/MAPbI_3_/Spiro-OMeTAD/Au flexible cells tested in this study. Cross-sectional SEM image of a completed device based on **(b)** rigid FTO glass substrate and **(c)** flexible ITO-PEN substrate.

**Figure 2:**
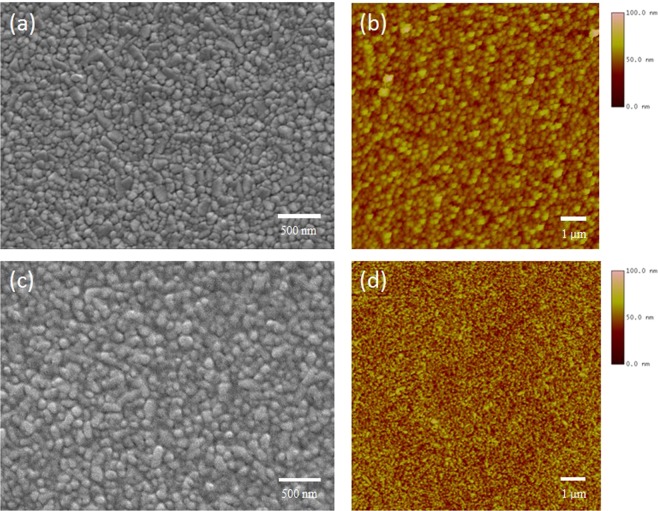
Comparison of sputtered SnO_2_ and spin-coated SnO_2_ films. (**a)** SEM and **(b)** AFM images of 40 nm sputtered SnO_2_ film. **(c)** SEM and **(d)** AFM images of spin-coated SnO_2_ film.

In this work, we demonstrated *n-i-p* planar structure of PSCs with optimized room-temperature-processed SnO_2_ as ETL prepared by RF magnetron sputtering and vacuum-deposited perovskite film. Figure 1a shows the schematic of device structure of our PSCs: ITO-PEN/SnO_2_/MAPbI_3_/Spiro-OMeTAD/Au. Figure 1b,c display SEM images of complete device based on rigid FTO glass substrate and flexible ITO-PEN substrate respectively. The morphology and surface roughness of bare FTO (Supplementary Fig. S1), 40 nm SnO_2_-coated FTO (Fig. 2a,b), and solution-processed SnO_2_ (Fig. 2c,d) were studied by SEM and atomic force microscopy (AFM). Supplementary AFM reveals the root-mean-square (RMS) roughness and mean roughness of bare FTO glass were 7.736 nm and 6.096 nm respectively. In contrast, 40 nm SnO_2_-coated FTO had a RMS roughness and mean roughness of 5.488 nm and 4.358 nm respectively, while solution-processed SnO_2_ had higher RMS roughness and mean roughness of 6.439 nm and 5.000 nm. It is reflected that sputtered SnO_2_ film was uniformly deposited on FTO surface and SnO_2_ grains were small enough to fill in the gaps between FTO grains, leading to a lower surface roughness than solution-processed SnO_2_, which is beneficial for the growth of vapor-deposited perovskite. To illustrate how surface roughness matters, perovskite was vapor-deposited on a 10 nm SnO_2_-coated FTO with RMS roughness and mean roughness of 7.199 nm and 5.708 nm respectively, measured by AFM (Supplementary Fig. S2). It was clear that perovskite grain sizes on the 10 nm SnO_2_-coated FTO were significantly reduced (Supplementary Fig. S3a). Moreover, cross-sectional SEM shown in Supplementary Fig. S3b reveals that the perovskite grains and shape became more irregular on 10 nm SnO_2_-coated FTO, while single-crystal-thick perovskite grains with larger grain sizes and regular shapes were crystallized on 40 nm SnO_2_-coated FTO (Supplementary Fig. S3c,d). In addition, small perovskite grains tended to crystallize in the perovskite-SnO_2_ interface on 10 nm SnO_2_-coated FTO, which could adversely affect the efficiency of carrier transport. Since materials were slowly deposited onto substrate down to a few angstroms per second, a rougher surface would hinder the ion migration and volumetric expansion as perovskite crystallization took place. In contrast, this problem becomes less significant when perovskite is prepared by solution methods because they allow ions within the precursors to easily spread all over the substrates without overcoming significant energy barriers. Therefore, this problem was not commonly discussed in depth before. In contrast, although perovskite grown on solution-processed SnO_2_ film (Supplementary Fig. S4) demonstrated comparable grain size with those grown on sputtered SnO_2_, the former perovskite films exhibit layered structure on most of the grains. This could be attributed to the higher roughness of solution-processed SnO_2_ film, leading to inconsistent rate and degree of perovskite crystallization and ultimately high surface roughness. These could increase the probability of carrier recombination between perovskite absorber and the HTL.

**Figure 3:**
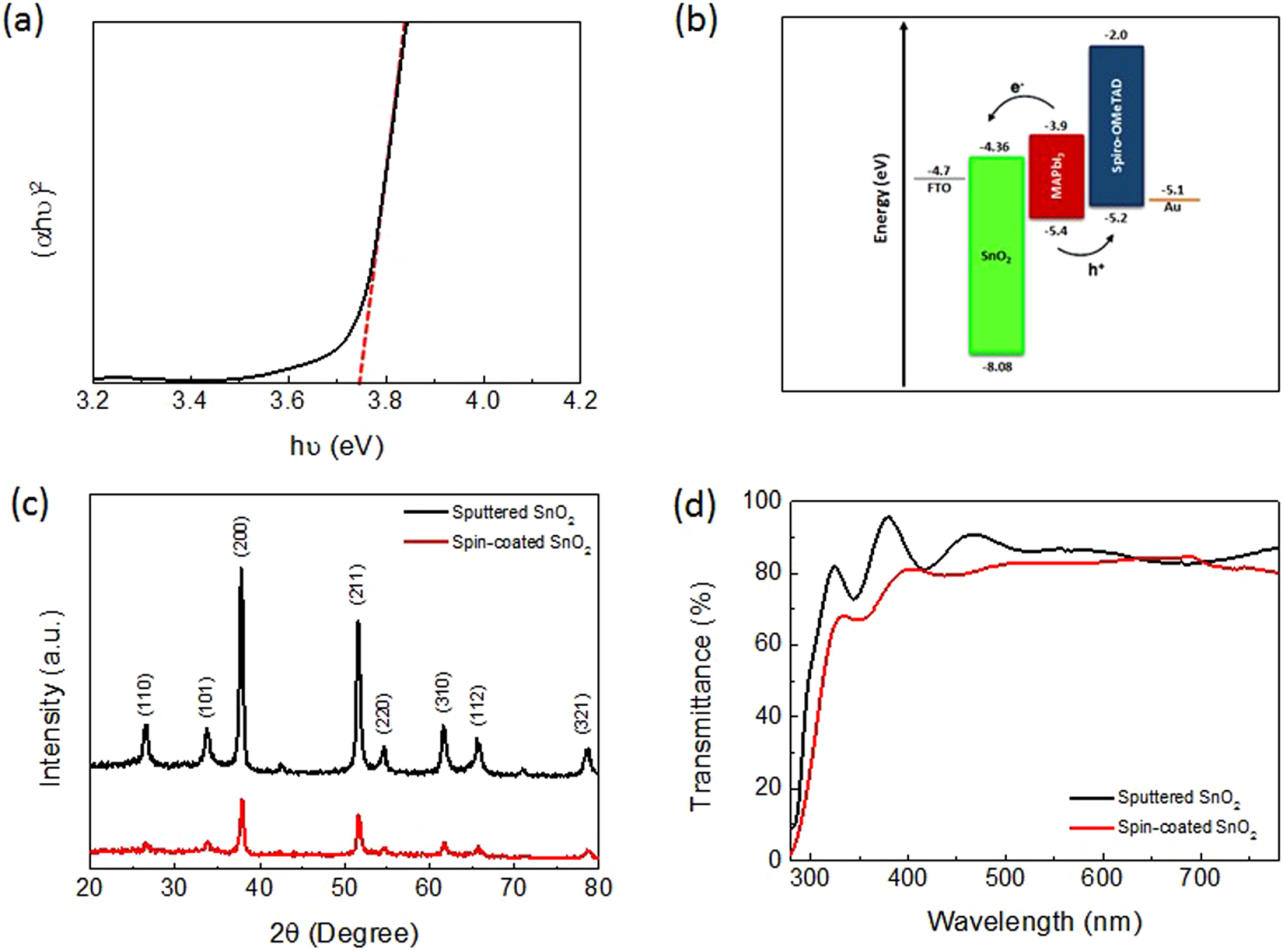
SnO_2_ thin film characterization. (**a**) Tauc plot of sputtered SnO_2_. (**b)** Energy levels (relative to vacuum) of various device components. **(c)** XRD and **(d)** transmittance spectra of sputtered SnO_2_ and spin-coated SnO_2_ films deposited on FTO glass.

The full ultraviolet photoelectron spectroscopy (UPS) spectrum of sputtered SnO_2_ is shown in Supplementary Fig. S5a. The sputtered SnO_2_ film showed a secondary cutoff edge of 5.01 eV (Supplementary Fig. S5b), indicating a work function W_S_ of 5.01 eV. Supplementary Fig. S5c shows the valence band maximum (VBM) of the sputtered SnO_2_ film is located at 3.07 eV, below E_F_. The bandgap of the sputtered SnO_2_ film acquired from the Tauc plot (Fig. 3a) was 3.72 eV, which is wider than that of ZnO and TiO_2_. A wider bandgap implies better hole blocking ability and can avoid absorption of high-energy photons which leads to small current loss^40^. Based on the above values, it can be calculated using the semiconductor band structure (E_C_ = W_S_ + VBM − E_g_) that the E_C_ of the sputtered SnO_2_ film was 4.36 eV, which is deeper than that of TiO_2_ and ZnO, both are 4.2 eV. The deeper conduction band of SnO_2_ compared to TiO_2_ and ZnO could enhance electron transfer from perovskite to the ETL. On the other hand, calculation showed E_V_ of the sputtered SnO_2_ film was 8.08 eV, which is much deeper than that of TiO_2_ and ZnO, 7.4 eV and 7.6 eV respectively. The deeper valence band of SnO_2_ can enhance hole blocking ability from perovskite to the ETL. Figure 3b shows the energy band diagram of each device component and the transportation of photo-generated electrons and holes.

Being an effective ETL for PSCs of *n-i-p* planar structure, transmittance, thickness, and film quality are crucial. XRD (Fig. 3c) revealed that both sputtered and solution-processed SnO_2_ films were polycrystalline but the former one exhibited better crystallinity. All XRD peaks for SnO_2_ were indexable to the tetragonal SnO_2_ structure, indicating the formation of pure SnO_2_ crystals. In addition, sputtered SnO_2_ film on FTO glass showed good transparency with transmittance close to 90% in the visible region (Fig. 3d), while solution-processed SnO_2_ had lower transmittance of about 80%. It is noteworthy the room-temperature sputtered SnO_2_ here demonstrated even higher transmittance than other high-temperature-processed spin-coated SnO_2_ films^41^. To obtain high-quality SnO_2_ films, impact of thickness, sputtering working pressure, and the flow rates of O_2_ and Ar during sputtering were systematically studied.

**Figure 4:**
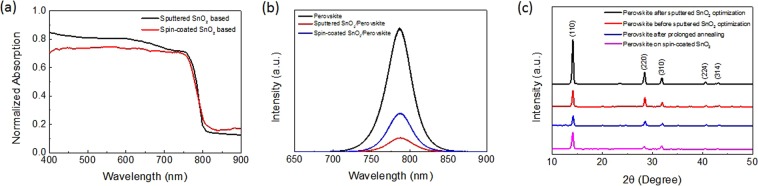
Properties of vapor-deposited perovskite film. (**a**) Ultraviolet-visible spectrum (UV-vis) of vapor-deposited MAPbI_3_ grown on sputtered and spin-coated SnO_2_ films. **(b)** Photoluminescence (PL) spectrum of vapor-deposited MAPbI_3_ deposited on sputtered and spin-coated SnO_2_ films. **(c)** XRD of vapor-deposited perovskite in different conditions.

Since the thickness of ETL can critically affect cell performance, SnO_2_ film was first sputtered under the same sputtering power of 60 W on FTO glass substrates kept at room temperature with four different thicknesses, 20 nm, 40 nm, 60 nm, and 80 nm, which took 8 min, 15 min, 23 min, and 30 min for sputtering, respectively. It can be inferred that the deposition rate was approximately 0.43 Å s^−1^. After sputtering, SnO_2_-coated FTO substrates were transferred to the evaporator for perovskite fabrication via a two-step vapor deposition as described in the Method section. The as-deposited perovskite samples were annealed in ambient air condition with over 65% humidity. It has been reported that certain level moisture is helpful for perovskite crystallization but excessive moisture could be detrimental to perovskite^42^. To overcome the humidity problem, perovskite samples were annealed for a short period of time at elevated temperature to accelerate the perovskite crystallization process and meanwhile minimize perovskite film degradation in ambient condition^39,43,44^. As-deposited perovskite samples were annealed at 130 °C for 10 min instead of the conventional 100 °C for an hour. It turned out that this method is also workable for vapor-deposited perovskite films, not only for solution-processed perovskite films. The UV-vis absorption spectra shown in Fig. 4a of the vapor-deposited perovskite shows good absorption in the visible region. It also revealed that perovskite grown on sputtered SnO_2_ exhibited higher absorption than that grown on solution-processed SnO_2_, which was attributed to its lower transmittance than sputtered SnO_2_. The absorption onset corresponded to an optical bandgap of 1.57 eV, estimated from the Tauc plot (Supplementary Fig. S6). The estimation matches well with the perovskite PL peak at 788 nm and the steady PL showed a more significant quench when depositing the perovskite film on sputtered SnO_2_ compared to solution-processed SnO_2_ (Fig. 4b). It supports that sputtered SnO_2_ possessed more efficient electron transport ability. XRD of perovskite (Fig. 4c) presented the expected perovskite pattern, with intense signals at 14.1°, 28.4°, and 31.9° corresponding to the (100), (200), and (310) directions, respectively. It showed an extra peak of PbI_2_ at 12.7° for perovskite that underwent 30 min prolonged annealing in humid air condition. Supplementary Fig. S7 shows the SEM of perovskite annealed for 30 min. The perovskite decomposing into PbI_2_ hindered its crystallization by grain boundary expansion and grain cracking. As a consequence, the intensity of each perovskite peak was clearly reduced as shown in Fig. 4c. It confirms the effectiveness of short-time annealing processing at elevated temperature in ambient condition.

**Figure 5:**
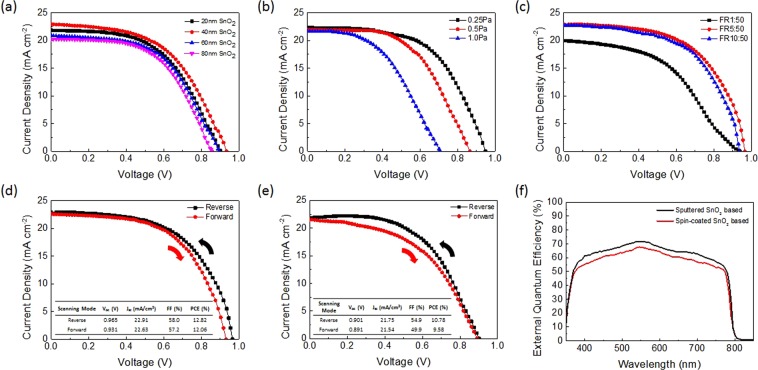
Device performance of perovskite solar cells based on sputtered SnO_2_ and solution-processed SnO_2_. *J-V* characteristics based on **(a)** different SnO_2_ thickness, **(b)** 40 nm SnO_2_ sputtered at different working pressures, and **(c)** 40 nm SnO_2_ sputtered at 0.25 Pa working pressure in different O_2_ to Ar flow rate ratios. *J-V* characteristics of the champion perovskite solar cell based on **(d)** optimized sputtered SnO_2_ and **(e)** solution-processed SnO_2_ measured under reverse and forward voltage scanning with AM1.5G illumination. **(f)** EQE curves of the champion devices based on sputtered and solution-processed SnO_2_ respectively.

The *J-V* characteristics of devices based on FTO glass substrates with different SnO_2_ thicknesses measured under AM1.5G illumination are shown in Fig. 5a. The device performance firstly increased and then decreased as the sputtered SnO_2_ thickness increased. It is seen that devices with 40 nm SnO_2_ yielded the best performance, with a PCE of 11.14%, a V_OC_ of 0.934 V, a J_SC_ of 22.91 mAcm^−2^, and a fill factor (FF) of 52.1%, so 40 nm was taken as the optimum thickness. If the SnO_2_ layer was too thin, it could not fully cover the FTO surface for effective electron transport. On the other hand, a too thick SnO_2_ layer would induce a larger series resistance.

The sputtering working pressure is another critical factor to determine the sputtered film quality. We fabricated PSCs based on 40 nm SnO_2_ sputtered under the same power of 60 W but three different working pressures, 0.25 Pa, 0.5 Pa, and 1.0 Pa. The *J-V* characteristics of respective devices are shown in Fig. 5b. The device performance, particularly the V_OC_, decreased as working pressure increased. It is seen that devices with SnO_2_ sputtered at 0.25 Pa yielded the best performance, with a PCE of 12.18%, a V_OC_ of 0.948 V, a J_SC_ of 22.34 mAcm^−2^, and an FF of 57.5%, so 0.25 Pa was taken as the optimum working pressure. An optimum working pressure is important so that the mean free path of gas molecules (O_2_ and Ar) is comparable to the distance between the target and substrates. A high working pressure will reduce the mean free path of molecules. In other words, there will be so much scattering that electrons will not have enough time to gather enough energy between collisions to ionize the atoms on the target. As a result, it causes a less uniform film deposition over the substrates. The reduced V_OC_ is attributed to uneven deposition due to too high working pressure. Sputtering under working pressure below 0.25 Pa was attempted, however, the plasma became unsustainable and unstable around 0.2 Pa due to too low gas molecule concentration. In order to yield a self-sustaining plasma, each electron has to generate enough secondary emission. Therefore, 0.25 Pa was concluded to be the optimum working pressure for SnO_2_ sputtering without compromising film quality and device performance.

The impact of flow rate of O_2_ and Ar during SnO_2_ sputtering on device performance was also studied. The flow rate of Ar was kept constant at 50 sccm while the flow rate of O_2_ was varied from 1 sccm, 5 sccm, to 10 sccm (defined as FR1:50, FR5:50, and FR10:50 respectively). Figure 5c shows the *J-V* characteristics of PSCs with 40 nm SnO_2_ sputtered under the same working pressure of 0.25 Pa but different flow rates of O_2_ and Ar. The FR5:50 (O_2_:Ar) PSC showed a PCE of 12.82% with V_OC_ of 0.965 V, J_SC_ of 22.91 mAcm^−2^, and FF of 58.0%. Its performance was slightly better that of the FR10:50 PSC. However, the FR1:50 PSC yielded the poorest performance: a PCE of 8.66% with V_OC_ of 0.938 V, J_SC_ of 19.97 mAcm^−2^, and FF of 46.2%. A possible reason is that too low oxygen flow rate (or partial pressure) does not favor stoichiometric SnO_2_ sputtering. The fewer O_2_ amount was not sufficient to compensate and combine with the tin ions sputtered out of the target to form high-quality SnO_2_ film and hence worsened the hole blocking ability. The performance of devices using SnO_2_ ETLs with different parameters are summarized in Table 1.

**Table 1:** Device performance of devices based on different sputtering parameters of SnO_2_.

SnO_2_ Sputtering Condition	V_OC_ (V)	J_SC_ (mAcm^−2^)	FF (%)	PCE (%)
20 nm	0.903	21.90	53.0	10.48
40 nm	0.934	22.91	52.1	11.14
60 nm	0.893	20.90	53.9	10.06
80 nm	0.855	20.26	54.9	9.51
40 nm 0.25 Pa	0.948	22.34	57.5	12.18
40 nm 0.50 Pa	0.865	21.93	55.0	10.44
40 nm 1.0 Pa	0.708	21.61	47.1	7.20
40 nm 0.25 Pa FR1:50	0.938	19.97	46.2	8.66
40 nm 0.25 Pa FR5:50	0.965	22.91	58.0	12.82
40 nm 0.25 Pa FR10:50	0.935	22.73	57.6	12.24

Figure 5d shows the *J-V* characteristics of the champion PSC using 40 nm SnO_2_ sputtered under optimized working pressure of 0.25 Pa and 5:50 O_2_:Ar ratio measured under reverse and forward scanning. It achieved a PCE of 12.82% with a V_OC_ of 0.965 V, a J_SC_ of 22.91 mAcm^−2^, and an FF of 58.0% when measured under reverse voltage scanning and a PCE of 12.06% with a V_OC_ of 0.931 V, a J_SC_ of 22.63 mAcm^−2^, and an FF of 57.2% when measured under forward voltage scanning. Therefore, the device exhibits a small hysteresis. In comparison, Fig. 5e shows the *J-V* characteristics of the champion PSC based on solution-processed SnO_2_. It achieved a lower PCE of 10.78% with a V_OC_ of 0.901 V, a J_SC_ of 21.75 mAcm^−2^, and an FF of 54.9% when measured under reverse voltage scanning and a lower PCE of 9.58% with a V_OC_ of 0.891 V, a J_SC_ of 21.54 mAcm^−2^, and an FF of 49.9% when measured under forward voltage scanning. It exhibited a more significant hysteresis. The external quantum efficiency (EQE) of both champion devices are shown in Fig. 5f.

To investigate the stability, both champion devices were left in room-temperature dry air with 30% humidity in dark for 192 hours. The PCE of each device was measured every 24 hours. It is found after 192 hours that the sputtered SnO_2_ based champion device could retain over 93% of its initial PCE while the solution-processed SnO_2_ based champion device could only retain 77% of its initial PCE. Supplementary Fig. S8 shows the *J-V* characteristics of both devices measured after 192 hours of stability test. The PCE of the sputtered SnO_2_ based device dropped to 11.91% with a V_OC_ of 0.929 V, a J_SC_ of 21.99 mAcm^−2^, and an FF of 58.3% while the PCE of the solution-processed SnO_2_ based device dropped to 8.34% with a V_OC_ of 0.853 V, a J_SC_ of 19.75 mAcm^−2^, and an FF of 49.5%. Supplementary Fig. S9a shows the evolution of their PCEs throughout the monitored period. The performance loss can be attributed to the degradation of the perovskite film within an unencapsulated device in 65% humidity. The highly hygroscopic and deliquescent properties of Li-TFSI used as dopant in Spiro-OMeTAD can also be regarded as a contribution to perovskite degradation due to penetration of water molecules^45^. In addition, the perovskite film could deteriorate rapidly in the presence of TBP as a polar solvent. Therefore, TBP may be a good solvent for perovskite, which means it can corrode the perovskite layer^46^. It is worth mentioning that the perovskite films, HTL films, and all devices were annealed, prepared, unencapsulated, and characterized in ambient condition with humidity greater than 65%. It convinces the viability of air processability of perovskite films, HTL films, and device characterization without the use of a glove box. To further illustrate this assertion, 40 sputtered SnO_2_ based PSCs in one single batch using the optimized SnO_2_ parameters and 40 solution-processed SnO_2_ based PSCs in one batch were fabricated by the same procedures. Statistics as shown in Supplementary Fig. S9b convince the reproducibility and consistency of device performance. Both batches of devices demonstrated normal distribution of PCEs. It can be concluded that sputtered SnO_2_ based devices showed higher average PCE and sputtered SnO_2_ is therefore more effective and beneficial to enhance PSC device performance and stability.

**Figure 6:**
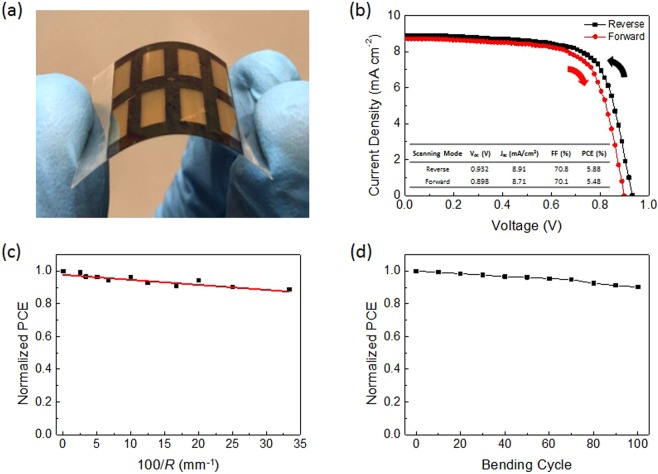
Photograph and device performance of a perovskite solar cell prepared on a flexible PEN substrate. (**a**) Photograph of PSCs prepared on a flexible ITO-PEN substrate. **(b)** *J-V* characteristics of the champion perovskite solar cell measured under reverse and forward voltage scanning with AM1.5G illumination. **(c)** Normalized PCE (measured on a flat surface) after bending the substrate with decreasing radii of curvature *R*. All measurements were performed on a single device from the highest radius of curvature to the lowest. The linear fit is provided as a guide to the eye. **(d)** Normalized PCE of a flexible PSC as a function of bending cycles at a radius of 2 cm.

Flexibility is a desirable feature of thin film solar cells for a variety of applications, such as portable power sources, building-integrated photovoltaics, clothing and textiles, power-generating fabrics, and electronics with light-weight curved surface. One of the main advantages in the use of sputtered SnO_2_ is that no sintering or annealing step is required thus flexible plastic substrates can be used. The other major fabrication steps (vapor deposition of perovskite, spin-coating of Spiro-OMeTAD, and thermal evaporation of Au) are also carried out in room temperature condition, which means this fabrication process as a whole is compatible with flexible substrates. To date, majority of the reported flexible PSCs employ spin-coated TiO_2_, ZnO, or PCBM as ETL^47–51^, while very few of them used solution-processed SnO_2_ as ETL. To illustrate the compatibility of sputtered SnO_2_ with flexible substrates for perovskite photovoltaic, in our work rigid FTO glass substrate was therefore replaced by flexible substrate, namely indium-doped-tin-oxide-coated polyethylene naphthalate (ITO-PEN) (Fig. 6a). Supplementary Fig. S10a shows the XRD spectrum of vapor-deposited perovskite grown on flexible ITO-PEN. It presented an expected perovskite spectrum with sharp signal intensities and without PbI_2_ residue, showing that vapor deposition and post-annealing treatment of perovskite on flexible substrates did not induce any perovskite degradation. 20 devices were fabricated using the same preparation procedures and their PCE distribution is summarized in Supplementary Fig. S10b. Figure 6b shows the *J-V* characteristics of the champion device on flexible ITO substrate. It yielded a PCE of 5.88% with a V_OC_ of 0.932 V, a J_SC_ of 8.91 mAcm^−2^, and an FF of 70.8% when measured under reverse voltage scanning and a PCE of 5.48% with a V_OC_ of 0.898 V, a J_SC_ of 8.71 mAcm^−2^, and an FF of 70.1% when measured under forward voltage scanning. Therefore, the device exhibits a small hysteresis. The discrepancies between J_SC_’s appeared in the studied devices measured under reverse and forward scannings might be the result of slow response of photocurrent and higher defect density^52^. It has been confirmed that higher defect density significantly contributes to the *J-V* hysteresis and degradation of photovoltaic parameters^52,53^. In comparison with the champion device based on rigid FTO glass substrate, the flexible devices yielded a substantial loss in J_SC_ but a significant improvement in FF. The loss of J_SC_ could be accounted to the lower transmittance through ITO-PEN substrates compared to FTO glass substrates and the threefold increase in device area from 3.14 mm^2^ to 10 mm^2^. On the other hand, the improvement in FF could be caused by the smoother ITO electrode surface, allowing flatter coverage of SnO_2_ film and eventually more compact interface between SnO_2_ film and perovskite for even more effective electron extraction and reduced series resistance.

Mechanical flexibility of devices under bending stress is of great importance concerning flexible and/or wearable device applications. Bending tests showed how well the device performance retained after being bent repeatedly to decreasing radii of curvature. The identical flexible PSC was bent by mechanical force with 10 different radii of curvature in one bending cycle. After each round of bending, the device performance was measured repetitively. The impact of bending on device PCE is presented in Fig. 6c. Less than 12% drop in PCE was observed. This result indicated sputtered SnO_2_ film is an effective and robust ETL for flexible PSC application. The impact of mechanical bending via multiple cycles of bending test was further evaluated. A total of 100 consecutive bending cycles at radius of 2 cm were performed. As shown in Fig. 6d, the device sustained over 90% of its initial PCE. After the bending test, V_OC_, J_SC_, and FF of the flexible device respectively dropped from 0.930 V to 0.929 V, from 8.76 mAcm^−2^ to 8.72 mAcm^−2^, and from 71.4% to 70.3%. Consequently, the PCE reduced from 5.82% to 5.69%. Although the flexible devices based on sputtered SnO_2_ demonstrated lower PCE than devices based on TiO_2_, ZnO, and PCBM, this is a pioneering work proving that sputtered SnO_2_ is effective and robust on both rigid and flexible substrates for perovskite photovoltaics. Sputtering technique is desirable for upscaling device area with uniform film deposition while flexible devices are especially attractive for a variety of consumer-driven products.

## Conclusion

We have pioneered the optimization and implementation of radio frequency magnetron sputtered SnO_2_ as electron transport layer for vapor-deposited-MAPbI_3_-based perovskite solar cells on both rigid and flexible substrates. It was demonstrated that neither mesoporous scaffold nor any high-temperature processing procedures were required to achieve efficient and air-stable devices without the use of a glove box. It is noteworthy that in the current device structure there was no backside passivation and all devices were not packaged, so the entire fabrication and characterization processes were subject to ambient condition of humidity greater than 65%. Despite the air processing in humid environment and perovskite annealing on flexible substrates, PSCs of 12.82% PCE on rigid glass substrates and 5.88% PCE on flexible substrates were achieved. We have also shown that the viability and repeatability of acquiring high-quality vapor-deposited perovskite films with large grain sizes and smooth morphology on sputtered SnO_2_ film via short-time annealing at elevated temperature processing in ambient condition, proving its compatibility with vapor-deposited perovskite films. More importantly, sputtered SnO_2_ based devices were demonstrated to have better device photovoltaic performance and stability than solution-processed SnO_2_ based devices. Such successful implementation of robust sputtered SnO_2_ films on flexible devices could serve as a promising route for future development and application of sputtered SnO_2_ film into large-scale cost-effective all-vacuum-deposited flexible perovskite photovoltaics.

## Methods

### Materials

FTO-coated glass substrates were purchased from Zhuhai Kaivo Optoelectronic Technology Co., Ltd. ITO-coated flexible PEN substrates were purchased from Peccell Technologies, Inc. The SnO_2_ target of 2-inch diameter was purchased from Chinese Rare Metal Co. Ltd. CH_3_NH_3_I (MAI) was purchased from Dyesol. PbI_2_, bis(trifluoromethane)sulfonimide lithium salt (Li-TFSI), 4-tert-Butylpyridine (TBP), and chlorobenzene were purchased from Sigma-Aldrich. N^2^,N^2^,N^2′^,N^2′^,N^7^,N^7^,N^7′^,N^7′^-octakis(4-methoxyphenyl)-9,9′-spirobi[9H-fluorene]-2,2′,7,7′-tetramine (Spiro-OMeTAD) was purchased from Lumtec. All materials were used as received.

### Device fabrication

The substrates were sequentially washed with acetone, isopropanol, and deionized water. The sheet resistance of FTO is 15 Ω □^−1^ and the thickness of glass and FTO are 1.6 mm and 420 nm respectively. The average transmittance of FTO glass in the visible region is 85%. The ITO-PEN has a sheet resistance of 15 Ω □^−1^, a thickness of 0.125 mm, and 78% transmittance in the visible region. SnO_2_ was deposited on FTO glass and ITO-PEN by radio frequency magnetron sputtering in room temperature. The clean substrates were transferred to a vacuum chamber and evacuated to a pressure of 4 × 10^−4^ Pa for SnO_2_ sputtering. The substrates were mounted on a rotating platform, 10 cm above the SnO_2_ target (China Rare Metal Co. Ltd.). The sputtering atmosphere was consisted of O_2_ and Ar. When 4 × 10^−4^ Pa was reached, O_2_ (99.99%) and Ar (99.99%) were pumped into the chamber. The gas flow rates of O_2_ and Ar were controlled by gas-flow meters and the gas flow ratio of O_2_ and Ar was set 1 sccm and 50 sccm, 5 sccm and 50 sccm, or 10 sccm and 50 sccm respectively. The working pressure for sputtering was maintained 0.25 Pa, 0.5 Pa, or 1.0 Pa. The SnO_2_ target was sputtered with a sputtering power of 60 W. The sputtered SnO_2_ thickness was set as 20 nm, 40 nm, 60 nm, or 80 nm at a deposition rate of 0.43 Å s^−1^. Solution-processed SnO_2_ films were prepared by spin-coating 0.1 M precursor solution of SnCl_2_ · 2H_2_O in ethanol at 3000 rpm for 30 seconds on clean FTO substrates. The SnO_2_ thin films were finally heated in air at 180 °C for 1 hour. The MAPbI_3_ perovskite was fabricated by a 2-step vapor deposition. The vapor deposition rate was controlled using a quartz sensor and calibrated after measuring the thickness of PbI_2_ and MAI films. The sources were located at the bottom of the chamber with an angle of 90° with respect to the SnO_2_-coated substrates. The distance between source and substrate was 20 cm. The evaporation rate of both PbI_2_ and MAI was maintained in a range of 1.5–2.0 Å s^−1^. 120 nm PbI_2_ and 280 nm MAI were evaporated to generate a resultant 400 nm MAPbI_3_ film. The as-deposited films were annealed at 130 °C for 10 minutes in ambient condition of 65% humidity. The perovskite films were then covered by Spiro-OMeTAD, which composed of 80 mgmL^−1^ chlorobenzene, 17.5 μL Li-TFSI (520 mgmL^−1^ acetonitrile), and 28.5 μL TBP, was spin-coated at 3000 rpm for 30 s. The films were left in a desiccator overnight. To complete the devices, 100 nm gold was deposited by thermal evaporation at 1 Å s^−1^ as an electrode. The device area on FTO glass and ITO-PEN were 0.0314 cm^2^ and 0.1 cm^2^, respectively.

### Device measurements

The AM1.5G solar spectrum was simulated by an Abet Class AAB Sun 2000 simulator with an intensity of 100 mWcm^−2^ calibrated with a KG5-filtered Si reference cell. The current-voltage (I–V) data were measured using a 2400 series sourcemeter (Keithley, USA). I–V sweeps (forward and reverse) were performed between −1.2 and +1.2 V, with a step size of 0.02 V and a delay time of 100 ms at each point.

### Material characterization

Field-emission scanning electron microscopy (JEOL JSM-7100F) and X-ray diffraction method (Bruker D8 X-ray diffractometer, USA) utilizing Cu K α radiation were used to study the thickness, morphology, roughness of the films, and phase characterization. The optical absorption and steady-state photoluminescence spectra were recorded on a Lambda 20 spectrophotometer (Perkin Elmer, USA) and InVia (Renishaw) micro raman/photoluminescence system, respectively. Ultraviolet photoelectron spectroscopy (Axis Ultra DLD) was used to determine the valence band maximum of SnO_2_ films. Scanning probe microscopy (NanoScope III) (Digital Instruments) was used to characterize the surface roughness of films.

## Supplementary information

Supplementary Information
