# Supplementary material for: Room-Temperature Sputtered SnO2 as Robust Electron Transport Layer for Air-Stable and Efficient Perovskite Solar Cells on Rigid and Flexible Substrates
Source: Sci Rep. 2019 May 6;9:6963. doi: 10.1038/s41598-019-42962-9 (PMC6502843; doi:10.1038/s41598-019-42962-9)
Supplement: Supplementary file 1 — Supplementary Information [file 41598_2019_42962_MOESM1_ESM.docx]

**Supplementary Information**

**Room-Temperature Sputtered SnO_2_ as Robust Electron Transport Layer for Air-Stable and Efficient Perovskite Solar Cells on Rigid and Flexible Substrates**

*Matthew Kam,^1,2^ Qianpeng Zhang, ^1,2^ Daquan Zhang, ^1,2^ Zhiyong Fan^1,2*^*

^1^HKUST-Shenzhen Research Institute, No. 9 Yuexing first RD, South Area, Hi-tech Park, Nanshan, Shenzhen 518057, China.

^2^Department of Electronic and Computer Engineering, Hong Kong University of Science and Technology (HKUST), Clear Water Bay, Kowloon, Hong Kong SAR, China.

*Corresponding to: eezfan@ust.hk


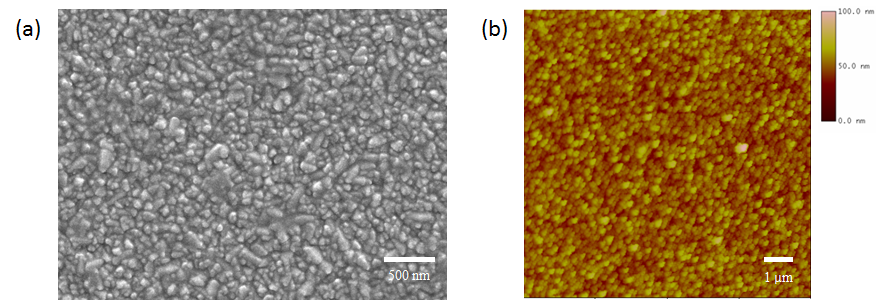


**Figure S1.** **(a)** SEM and **(b)** AFM of a FTO glass substrate.


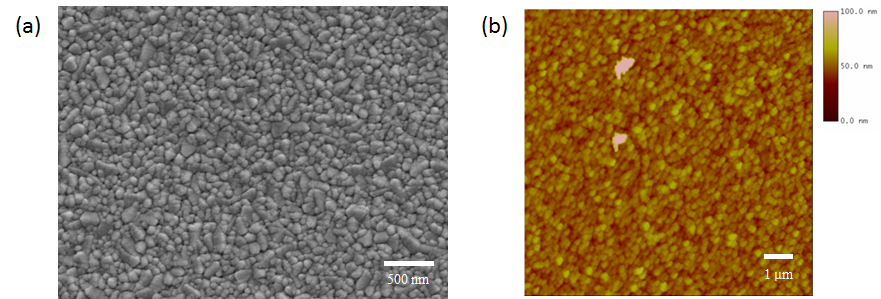


**Figure S2.** **(a)** SEM and **(b)** AFM of a FTO glass substrate coated with 10 nm sputtered SnO_2_.


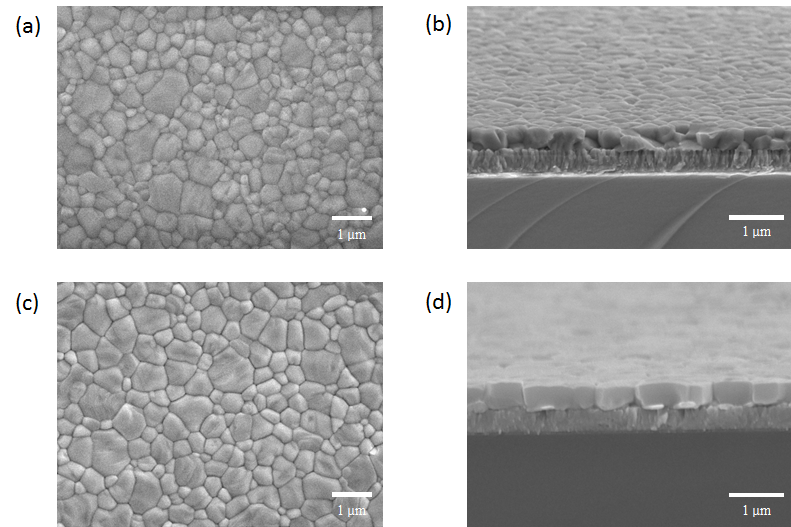


**Figure S3.** SEM of vapor-deposited perovskite on **(a-b)** 10 nm and **(c-d)** 40 nm SnO_2_-coated FTO.


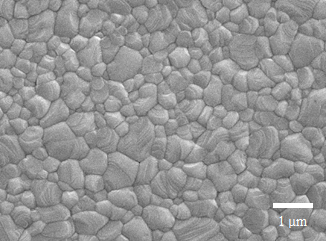


**Figure S4.** SEM of vapor-deposited perovskite grown on spin-coated SnO_2_ film.


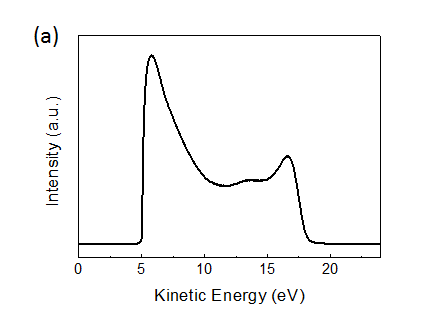


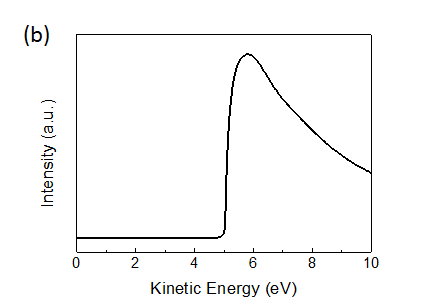


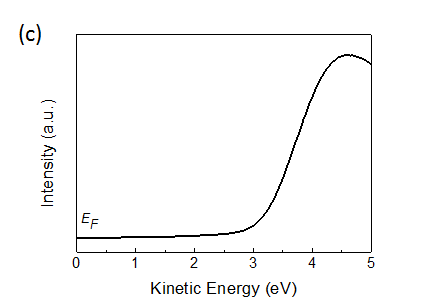


**Figure S5.** **(a)** Full ultraviolet photoelectron spectroscopy (UPS) spectrum of sputtered SnO_2_. **(b)** Cutoff edge of SnO_2_. **(c)** Valence band spectrum of SnO_2_.


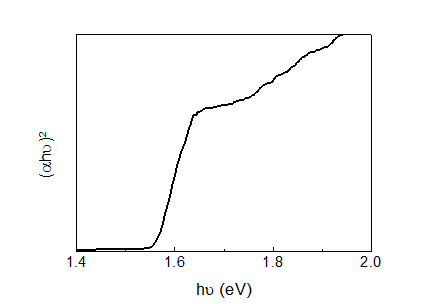


**Figure S6.** Tauc plot of $\mathrm{MAPbI}_{3}$ perovskite.


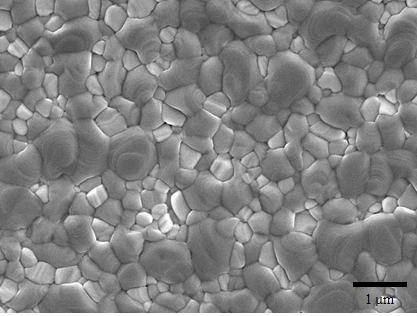


**Figure S7.** Vapor-deposited perovskite after 30 min prolonged annealing in ambient air.


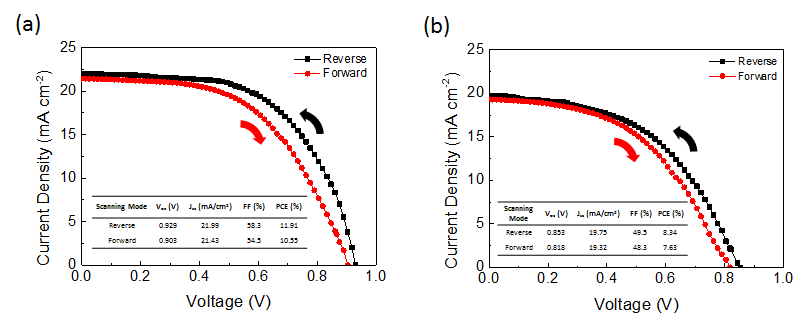


**Figure S8.** *J-V* characteristics (reverse scan and forward scan) of the champion perovskite solar cell on **(a)** rigid FTO glass substrate and **(b)** flexible ITO-PEN substrate 192 hours after fabrication.


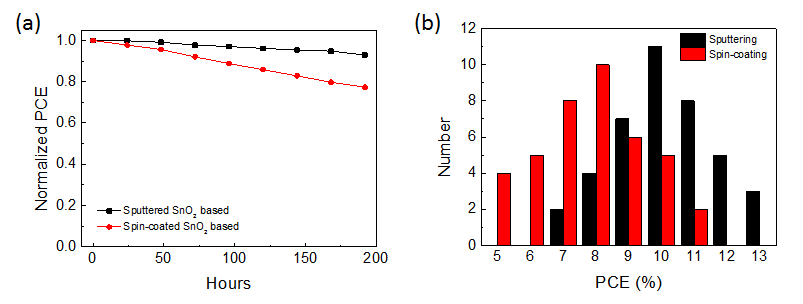


**Figure S9.** **(a)** 192-hour stability test of the champion perovskite solar cell based on sputtered and solution-processed SnO_2_ films respectively. **(b)** PCE distribution of 80 devices, containing one batch of 40 devices based on sputtered SnO_2_ and another batch of 40 devices based on solution-processed SnO_2_.


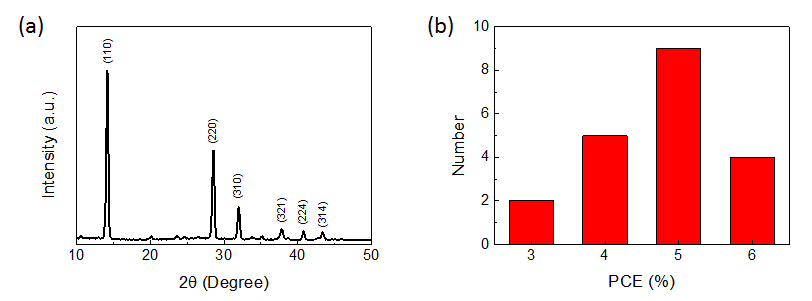


**Figure S10.** **(a)** XRD spectrum of vapor-deposited perovskite grown on flexible ITO-PEN substrate. **(b)** PCE distribution of one batch of 20 flexible devices based on sputtered SnO_2_.
